# Echo state networks for modeling turbulent convection

**DOI:** 10.1038/s41598-024-79756-7

**Published:** 2024-12-02

**Authors:** Mohammad Sharifi Ghazijahani, Christian Cierpka

**Affiliations:** Institute of Thermodynamics and Fluid Mechanics, Technische Universität Ilmenau, Ilmenau, 98684 Germany

**Keywords:** Echo state networks, Turbulence, Rayleigh-Bénard convection, Reduced order modeling, Fluid dynamics, Computer science

## Abstract

Turbulent Rayleigh-Bénard convection (RBC) is one of the very prominent examples of chaos in fluid dynamics with significant relevance in nature. Meanwhile, Echo State Networks (ESN) are among the most fundamental machine learning algorithms suited for modeling sequential data. The current study conducts reduced order modeling of experimental RBC. The ESN successfully models the flow qualitatively. Even for this highly turbulent flow, it is challenging to distinguish predictions from the ground truth. The statistical convergence of the ESN goes beyond the velocity values and is represented in secondary aspects of the flow dynamics, such as spatial and temporal derivatives and vortices. Finally, ESN’s main hyperparameters show values for best performance in strong relation to the flow dynamics. These findings from both the fluid dynamics and computer science perspective set the ground for future informed design of ESNs to tackle one of the most challenging problems in nature: turbulence.

## Introduction

Various disciplines are confronted with chaotic systems. Turbulence is a prominent and relevant example, where, although the boundary conditions and governing equations are well-defined, the presence of nonlinearity results in the unpredictability of small and large-scale dynamics of a flow. This is where Machine Learning (ML) steps in with the promise that a group of connected artificial neurons is able to model such complex dynamics without solving the underlying physical equations, which is often too costly in terms of computer power. In this regard, the current study aims for reduced-order modeling of Rayleigh-Bénard Convection (RBC) as one of the ultimate examples of turbulence by Echo State Networks (ESN) as one of the most basic and computationally very efficient ML algorithms.

**Fig. 1:**
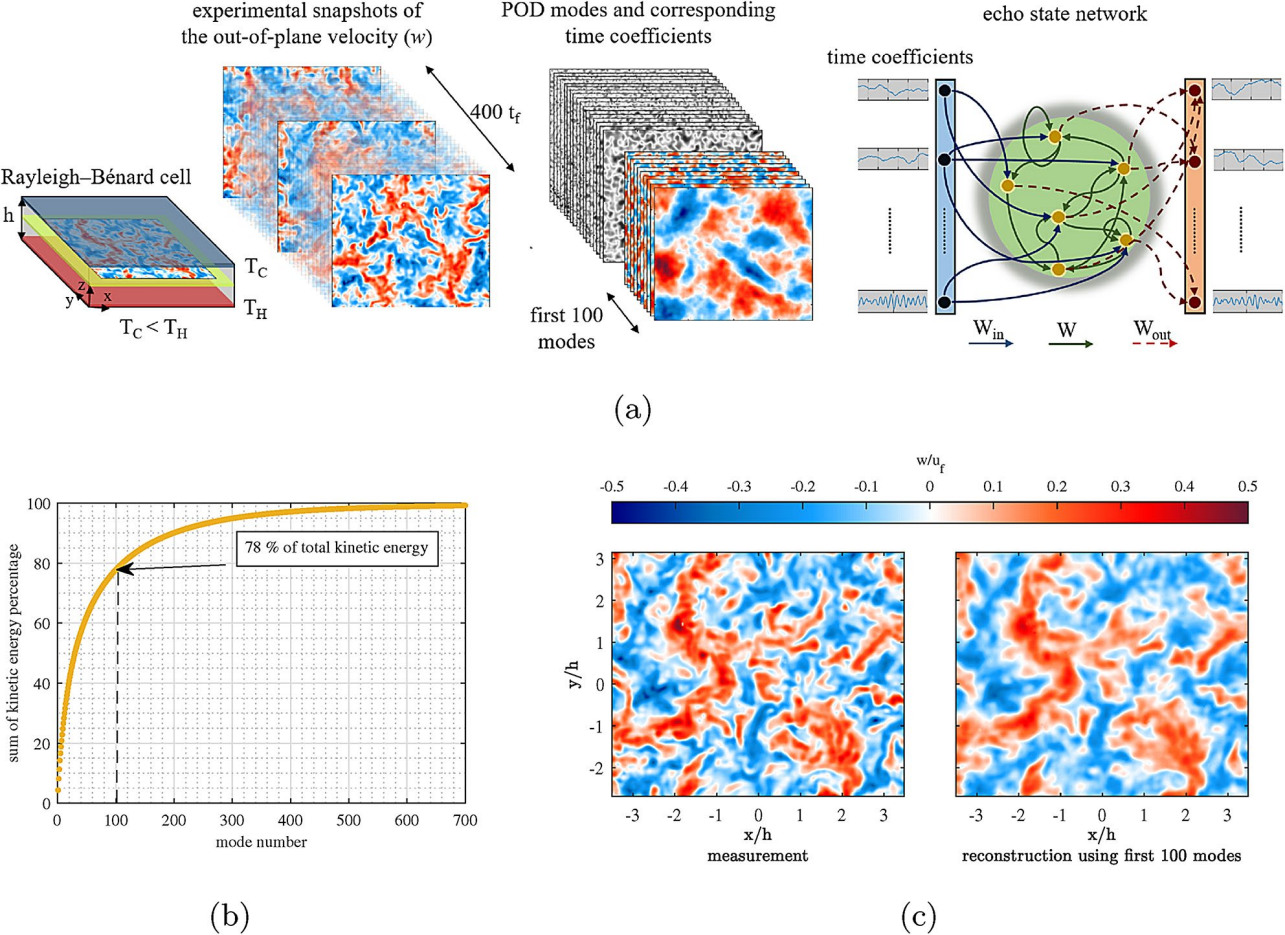
**a** Schematic of the applied methodology in the study. **b** Sum of kinetic energy percentage of POD modes for 400 $t_f$. **c**, The out-of-plane velocity component (*w*) of the fully resolved field vs the approximated field by the first 100 most energetic modes. Figures were generated using MATLAB R2020b (www.mathworks.com/products/new_products/release2020b.html ) and PowerPoint 2016 (www.microsoft.com/de-de/microsoft-365/powerpoint?market=de) softwares.

RBC is the buoyancy-driven motion in a fluid layer of height *h* confined between a heating plate below, a cooling plate above, and adiabatic side walls (see Fig. 1a). It is often referred to as the drosophila of turbulence due to its rich dynamics and significant relevance. Turbulent convection is characteristic for various natural phenomena on the earth’s mantle^1^, atmosphere^2^, oceans^3^, and stars^4^, and therefore, any advance in its modeling will improve our understanding of nature, for example, climate. Although all these examples are highly three-dimensional problems in large aspect ratio domains, initial studies on two-dimensional (2D) systems have been more prevalent due to the simpler dynamics of the flow. Pandey and Schumacher^5^were among the first to apply ML for modeling the velocity field in 2D RBC. Later, the study was expanded to moist 2D RBC^6^. The general outcome of these studies^7–9^is that ML is capable of modeling the much simpler dynamics of turbulent RBC for 2D cells. Meanwhile, instead of the reconstruction of the full velocity field, some investigations have aimed for some secondary, less challenging, yet valuable parameters in the 2D RBC, such as the modeling of heat and mass flux^10–12^, or conducting super-resolution^13^, and flow control^14,15^. Similar endeavors have been continued in 3D RBC for modeling the heat transfer^16^, the onset of convection^17^, extreme vortices^18^, and assimilation of the velocity field from the temperature field and vice versa^19,20^.

The ultimate challenge is the reduced order modeling of the complete velocity field in a large aspect ratio 3D highly turbulent RBC that so far has not been addressed. Besides, most of the previous studies in less complex systems have relied on open-loop modeling, where a significant amount of information on the ground truth is available for the network in the prediction phase. Furthermore, also the application of complex deep learning algorithms for modeling of chaotic systems result in favorable outcomes^21–23^, however, one can claim that simpler approaches might be more in line with the general claim of machine learning that some randomly connected nodes can actually learn and imitate chaotically behaving systems. In this regard, ESNs are viable candidates with proven capability in dealing with turbulent flows^5,6,24–26^ while maintaining a simple structure in general. An ESN is a reservoir of sparsely, randomly connected neurons (see Fig. 1a). Only the output layer is trained^27,28^, and the internal connection of each neuron with its previous state creates a kind of internal memory, an architecture ideal to learn from time series. Unlike many early studies, where perfect numerical simulations served as the ground truth, we will apply the ESN to experimental data. Thus, despite the benefit of such numerical methods in describing reality with all (but often unknown) boundary conditions, the observables (here being velocity data) are subjected to uncertainties due to the experimental methods.

## Problem set-up

The current study aims at reduced order modeling of the turbulent flow in the horizontal mid-plane of an RBC with an aspect ratio (width/height) of 10 using an ESN in a closed-loop scenario (see Fig, 1a). The flow data is from stereoscopic laser-optical experiments for a Rayleigh number of Ra = $5.5\times 10^6$ and a Prandtl number of Pr = 0.79 in ${\hbox {SF}}_6$(see methods for further information). A detailed discussion about the flow dynamics can be found in Ghazijahani and Cierpka^29^. One can generally describe the flow as highly turbulent, which constantly evolves within so-called turbulent superstructures^30^that never repeat in the future (see Ghazijahani and Cierpka^29^). This is evident from the Proper Orthogonal Decomposition (POD) results. POD is a data reduction method that reduces flow data to a set of spatial modes and respective time coefficients optimal w.r.t. to kinetic energy^31^. This approach effectively emphasizes the main flow characteristics in a more streamlined manner. Figure 1b shows the sum of the kinetic energy percentage of POD modes for 400 free-fall times ($t_f$ a characteristic timescale, see Sec. 5), which corresponds to 1400 snapshots. In this condition, the first 100 POD modes represent 78 % of the total kinetic energy. Figure 1c shows a comparison between the flow fields of the fully resolved measurements and the approximated flow by the first 100 POD modes. It is clear that the main features of the flow are preserved in the approximation. Therefore, the ESN is going to be trained for 200 $t_f$ (700 snapshots) by the Time Coefficients of POD Modes (TCPM) of the first 100 modes and then predict (model) the remaining 200 $t_f$ in a closed loop scenario. Closed loop in this respect means that the results serve as the predecessor for the new predictions. Here, one concern is that the current model may be difficult to apply to unseen parameters of Ra and Pr as POD is first used to reduce the input data dimension. In this regard, the reason for using POD modes instead of the velocity values is that the large-scale motions have an underlying structure represented by the POD modes, which are more accurately captured when the dataset (i.e., the duration of the snapshots) is sufficiently large. As the Rayleigh number (Ra) varies significantly, the characteristic size of these structures changes, rendering the existing basis invalid and requiring the construction of new POD modes. However, this also applies to the velocity fields, which would need to be updated accordingly. Three main hyperparameters of the ESN, namely leaking rate (LR), spectral radius (SR), and input scaling (INS), along with the random initialization of the network state (random seed (RS)), are varied to find the best set of hyperparameters for modeling. The optimization has been conducted based on the Weighted Prediction Score (WPS). Detailed information about the network, optimization, and experiments is available in Sect. 5.

## Results

**Fig. 2:**
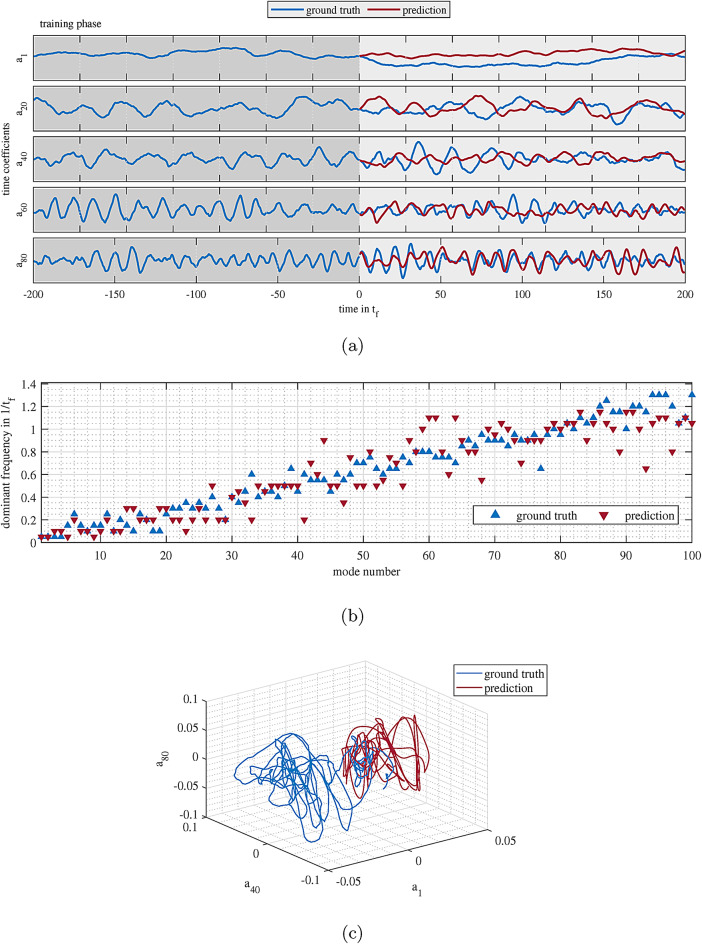
**a** Ground truth and prediction of the temporal coefficients of modes 1, 20, 40, 60, 80. The predictions are for the network with the optimized set of hyperparameters with INS = 20, SR = 0.5, and LR = 0.1. The first half of the data with the gray background shows the time steps that are used for the training of the ESN. **b** The dominant frequencies of time coefficients of the first 100 POD modes for the ground truth data and the predictions. **c** The three dimensional plot of the $a_1$, $a_{40}$, and $a_{80}$ variations with respect to each other for the ground truth and prediction. Figures were generated using MATLAB R2020b software (www.mathworks.com/products/new_products/release2020b.html).

**Fig. 3:**
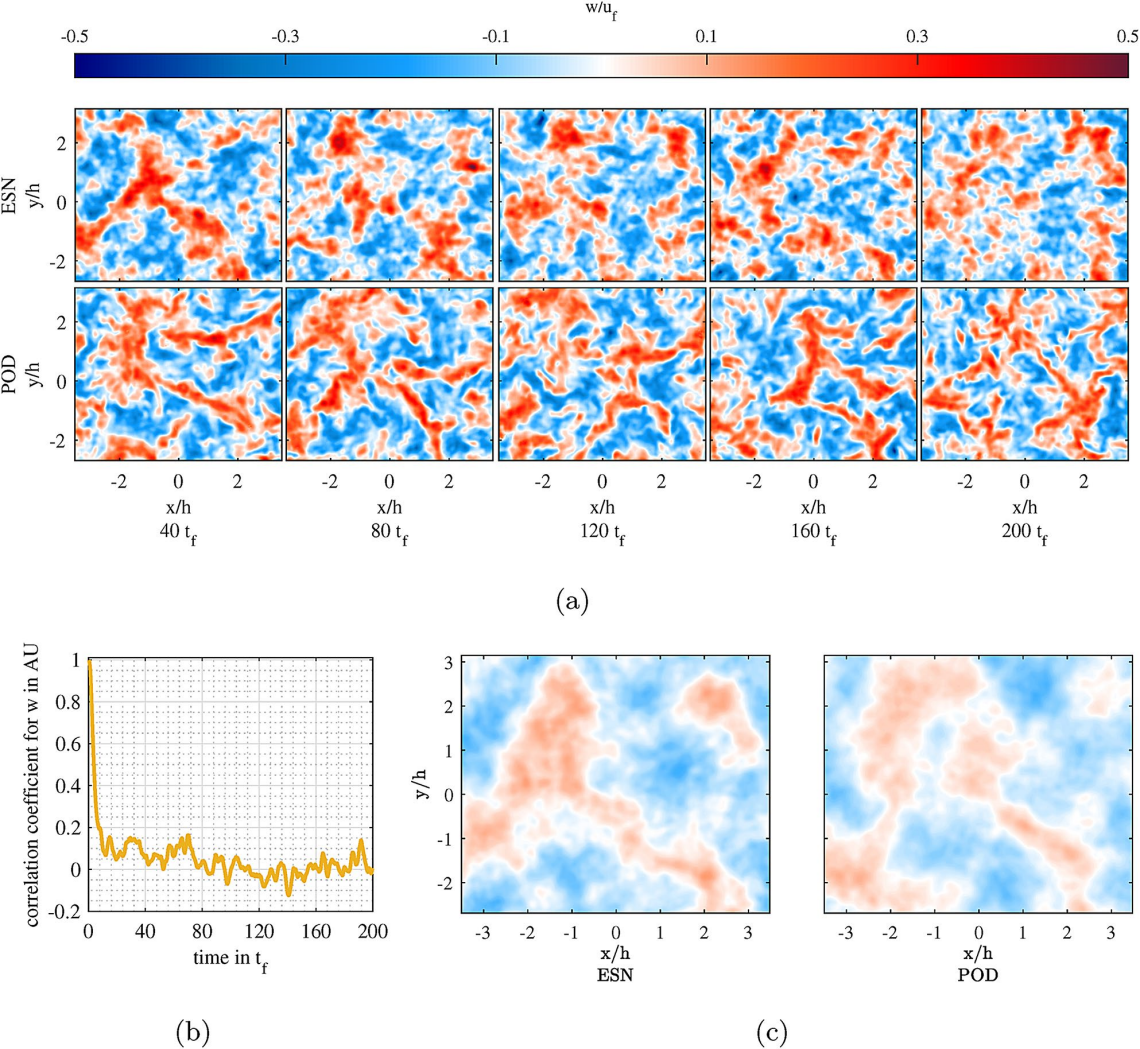
**a** Ground truth and reconstructed predictions of the out-of-plane velocity (*w*) field. **b** The correlation coefficient between the ground truth and reconstructions. **c** Corresponding average out-of-plane velocity (*w*) field in the entire prediction phase. Figures were generated using MATLAB R2020b software (www.mathworks.com/products/new_products/release2020b.html).

**Fig. 4:**
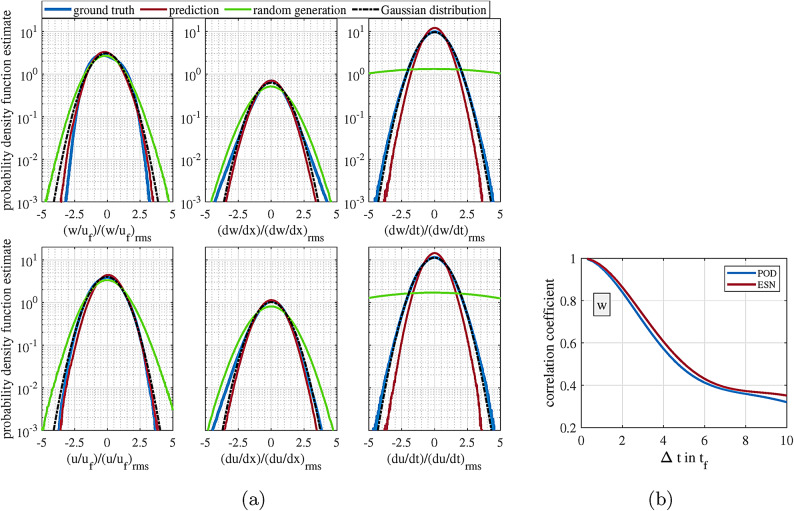
**a** Probability density function estimate (PDF) of the out-of-plane velocity (*w*), spatial derivative of the out-of-plane velocity (*dw*/*dx*), and temporal derivative of the out-of-plane velocity (*dw*/*dt*) for the ground truth, prediction, randomly generated data, and Gaussian distribution with similar mean and standard deviation to the ground truth at the top, and parallel values for one of the in-plane velocity components *u* at the bottom. **b** Correlation decay of out-of-plane velocity (*w*) for the ground truth and prediction. Figures were generated using MATLAB R2020b software (www.mathworks.com/products/new_products/release2020b.html).

### Predictions

Figure 2a shows the TCPMs of the ground truth and the predictions. The first 200 $t_f$ with the gray background shows the training phase, and the second half represents the prediction phase. Clearly, the network is faced with a combination of modes with very complex temporal evolution and superposition. As lower modes in this flow are typically related to larger structures, their temporal evolution is slow. However, the higher modes represent turbulent fluctuations on smaller scales and show almost periodic oscillations around zero with higher frequencies. To predict their combinations to resemble the highly turbulent flow is a significant challenge for the rather simple ESN (see Fig. 2b). In the prediction phase, therefore, the goal is to have a model of the flow that can mimic these dynamics without aligning with the ground truth deterministically. It may even be debated if the deterministic prediction of a turbulent flow might be possible at all. The red lines in Fig. 2a represent the reconstruction by the ESN for the optimized hyperparameter set based on maximum WPS (see Sec. 5 for further details) of 24 random seeds (INS = 20, SR = 0.5, and LR = 0.1). One can claim that the reconstructions are successful in grasping the dynamics of the system. In other words, if the prediction and ground truth had been plotted with the same color, picking the right one by eye would have been challenging as typical structure size and spatial distribution are well resembled. Furthermore, the predictions are very resilient against divergence, and they reliably stay in the relevant range, showing typical oscillations even at the very last stages of the prediction. This indicates the stability of the trained system inside the reservoir in the time domain even in this closed loop scenario where the current prediction serves as starting point for the next time step. Finally, from Fig. 2b, it is apparent that most of the predictions have a dominant frequency in the same range of ground truth, although some outliers exist. Figure 2c shows a 3D plot of the $a_1$, $a_{40}$, and $a_{80}$ in the entire prediction period for the ground truth and prediction. Clearly, the interdependencies between the modes are very complex, however, one can intuitively realize that the prediction show a similar type of interdepencies as well. Perhaps the only real difference is that in the ground truth the first mode is oscillating in the positive domains while the prediction stays at the opposite side.

POD modes and their respective TCPMs are rooted in the velocity field; thus, the reconstructed velocity field is the main point of concern, which reveals whether the ESN is able to grasp the interdependencies of the TCPMs to reconstruct reasonable dynamics in the velocity field. Nevertheless, quantifying the degree of similarity between the dynamics of the reconstructed model and the ground truth is necessary to determine whether the ESN is successful and whether it could be a tool in subgrid modeling or turbulent flow prediction. Figure 3a shows the reconstructed out-of-plane velocity (*w*) fields by the ESN vs the ground truth (POD) in the prediction phase. Even though all three components of velocity are reconstructed by the ESN, *w* is the main driver of the convection-dominated flow in the RBC, so it will be the focus of the analysis. The general comparison of the *w* fields in a very intuitive manner reveals that the reconstructions can be attributed to the same type of dynamics that are deriving the actual flow. In Fig. 3b, the correlation coefficient of the modeled velocity field with the ground truth is shown. It can be clearly seen that the modeling is not deterministic, as the correlation coefficient drops fast after already 10 $t_f$ after the training phase. For longer time spans in the closed-loop model the reconstructions are not anymore correlated to the ground truth and the model constructs a different but reasonable realization of the turbulent flow evolution. Figure 3c shows the average *w* field for the entire 200 $t_f$ in the prediction phase. Here also, a similar range of values (approximately $\pm 0.1$) and similar shapes and distributions of *w* field can be attributed to the reconstructions and ground truth.

In Fig. 4a top left, the Probability Density Function (PDF) estimates of the *w* field for the entire prediction duration are compared with the ground truth. As RBC in the mid-plane is dominated by upwelling hot and downwelling cold fluid, the PDF shows the typical broad but symmetric shape with zero mean value. Apparently, the PDFs of the model are well in line with the ground truth, although the extreme values are slightly overrepresented. This is an indication of the capability of ESNs in realizing the fact that there must be a balance between upward and downward flows in the field. This is quite spectacular when one considers that the POD modes, especially the most energetic ones, have very non-uniform shapes with complex oscillations of their TCPMs with respect to each other. Nevertheless, ESN is able to produce similar dynamics of the TCPMs, which, when combined to reconstruct the velocity field, result in general statistical agreement with the ground truth. Here, one might argue that the nature of the POD modes is in a way, that any set or combination of them will hold the statistical similarity with the ground truth. In order to counter this argument, the PDF of randomly generated TCPMs for similar 700 time steps is shown in the figure as well. It should be noted that the random TCPMs have been set to have the same mean and standard deviation as the ground truth, yet as can be seen, significant divergence from ground truth is visible. The next point of concern is the shape of the plumes in the field. This can be represented by the spatial derivatives of the velocity values (*dw*/*dx* or *dw*/*dy*) in the field. Although the shapes seem very arbitrary at first glance, they have a certain statistical distribution, as can be seen in Fig 4a middle. Evidently, the ESN has also represented this to some degree in the reconstructed field, although this time, extreme values are a bit underrepresented in the predictions. Similar graphs are represented for one of the in-plane velocity components (*u*) in Fig. 4a bottom, and the general conclusions are basically the same as the case of *w*. Furthermore, in each case the Gaussian distribution with similar standard deviation and mean as the case of ground truth is shown for the sake of comparison.

Finally, the speed of the variation in the *w* field (*dw*/*dt*) is also an important flow feature. In Fig. 4a right, it is clear that there is a general statistical alignment in this regard as well. Whereas for the spatial distribution, a random pick of the TCPMs gives results that do not deviate too much, the temporal evolution can only be modeled by the ESN. This is important if such methods shall be used for the subgrid modeling of large low-resolution simulations like weather or climate models. Nevertheless, such tasks will differ considerably from the one at hand in terms of their nature. However, fast shifts in *w* values tend to be slightly underrepresented in the predictions by the ESN. In this regard, the temporal derivative of *w* is a more local measure in general, and in order to quantify the speed at which previous structures in the flow are being replaced with the new ones, one needs to take a look at the average correlation decay in the *w* field as shown in Fig. 4b. Previously, it has been shown (see Ghazijahani and Cierpka^29^) that the trend of this correlation decay is an important feature of the RBC flow, which has a direct relation with the Rayleigh number in the cell (see Sec. 5 for definition). It is evident from the figure that the ESN has been able to imitate the speed of correlation loss in the flow very well, which is again very important when one considers the complexity of the spatial distributions of the POD modes and the oscillations of their TCPMs in time and the fact that this correlation loss is a variable that needs to be imitated in the combination of all reconstructed modes by the ESN. Last but not least, dissipation rate of turbulent kinetic energy $\epsilon$ is a main indicator of level of turbulence in the flow which can identify the general dynamics of the small eddies in the flow (see Eq. 1, $\nu$ is the kinematic viscosity)^32^. However, the problem is that in the current measurement only the in-plane derivatives of the velocity components along with the $\partial w/\partial z$ (from continuity equation of incompressible flows) are available and $\partial v/\partial z$ and $\partial u/\partial z$ can not be computed and are therefore simply set to zero. However, the $\epsilon$ can still reveal valuable insight about the similarity of prediction in terms of the dissipation rate in the flow. With this criteria, the prediction and ground truth have $\epsilon = 1.7$ and $2.1 \times 10^{-5}$ ${\textrm{m}}^{2}/{\textrm{s}}^{3}$, respectively. This shows that the turbulence dissipation rates are in the same order of magnitude with prediction having roughly 20 % less value, which is a further proof of general similarity of the prediction and ground truth. This was expected as the PDF of the gradients show a higher share of smaller values.1

### Vortices

**Fig. 5:**
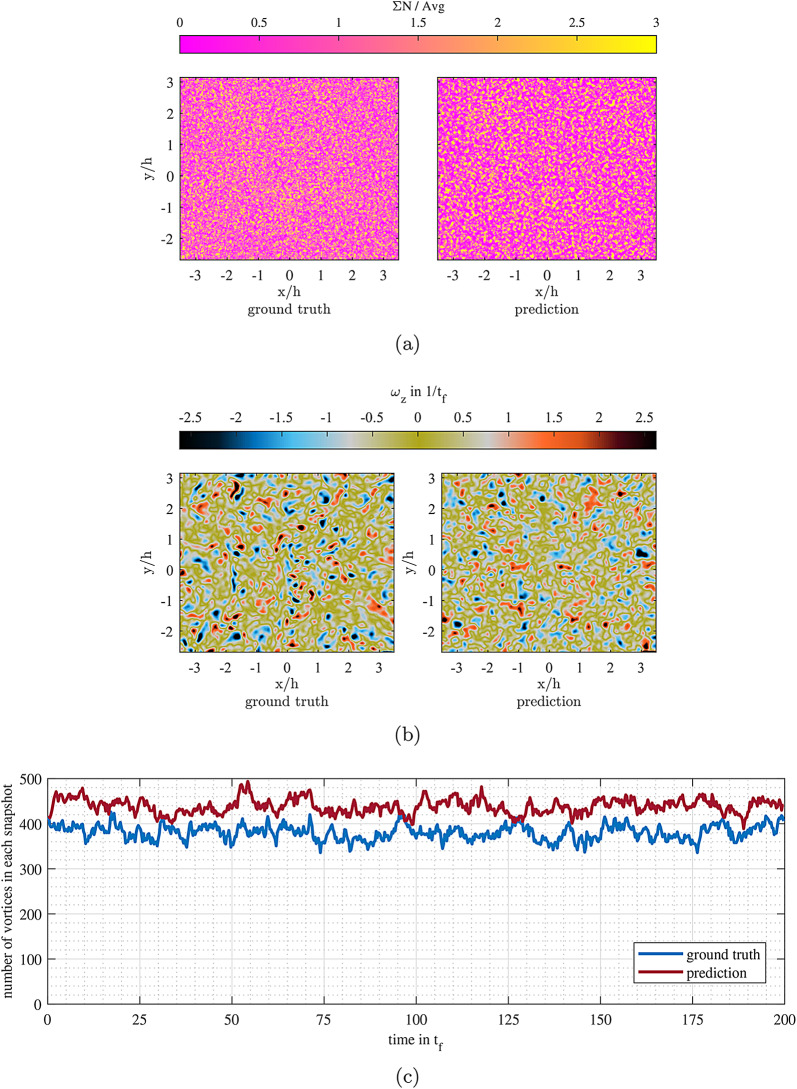
**a** Sum of the number of vortex centers in each grid point ($\Sigma N$) divided by the average number of them for the entire field for the entire duration of prediction for the ground truth and prediction. **b** Instantaneous out of plane vortex fields in the middle of prediction phase. **c** The number of out-of-plane vortices with respect to time for ground truth and prediction. Figures were generated using MATLAB R2020b software (www.mathworks.com/products/new_products/release2020b.html).

Out-of-plane vortices are among the main features of RBC. They are associated with the detachment of the plumes from thermal boundary layers in the top and bottom plates and are one of the main ways for energy dissipation in the flow. They have been perceived as one of the main indicators of the shift from soft to hard turbulent convection^33,34^. This raises the question of why these vortices are not used to train the ESN if they are so crucial to the RBC flow, and the answer is that due to their high speed dynamics and short life time, it is difficult to consider them as input information for the network to train and model for an extended period of time. Previously, it has been shown that the appearance of these vortices in the flow are completely random, and there are no preferred regions for them^29^. Figure 5a and 5b show that the ESN model features similar vortex distribution, counts, and size. The total number of these vortices remains almost constant in time, which is proportional to the flow’s Rayleigh number. As can be seen from Fig. 5c, the prediction also represents a quite constant number of vortices in their snapshots with a similar type of fluctuations as the ground truth; however, the number always stands a bit higher for the ESN. Assuming that the presence of these vortices is the main source of distortions in the flow, Fig. 5 proves that there are indeed clear rules in the background that ESN is successfully following.

### Hyperparameters

**Fig. 6:**
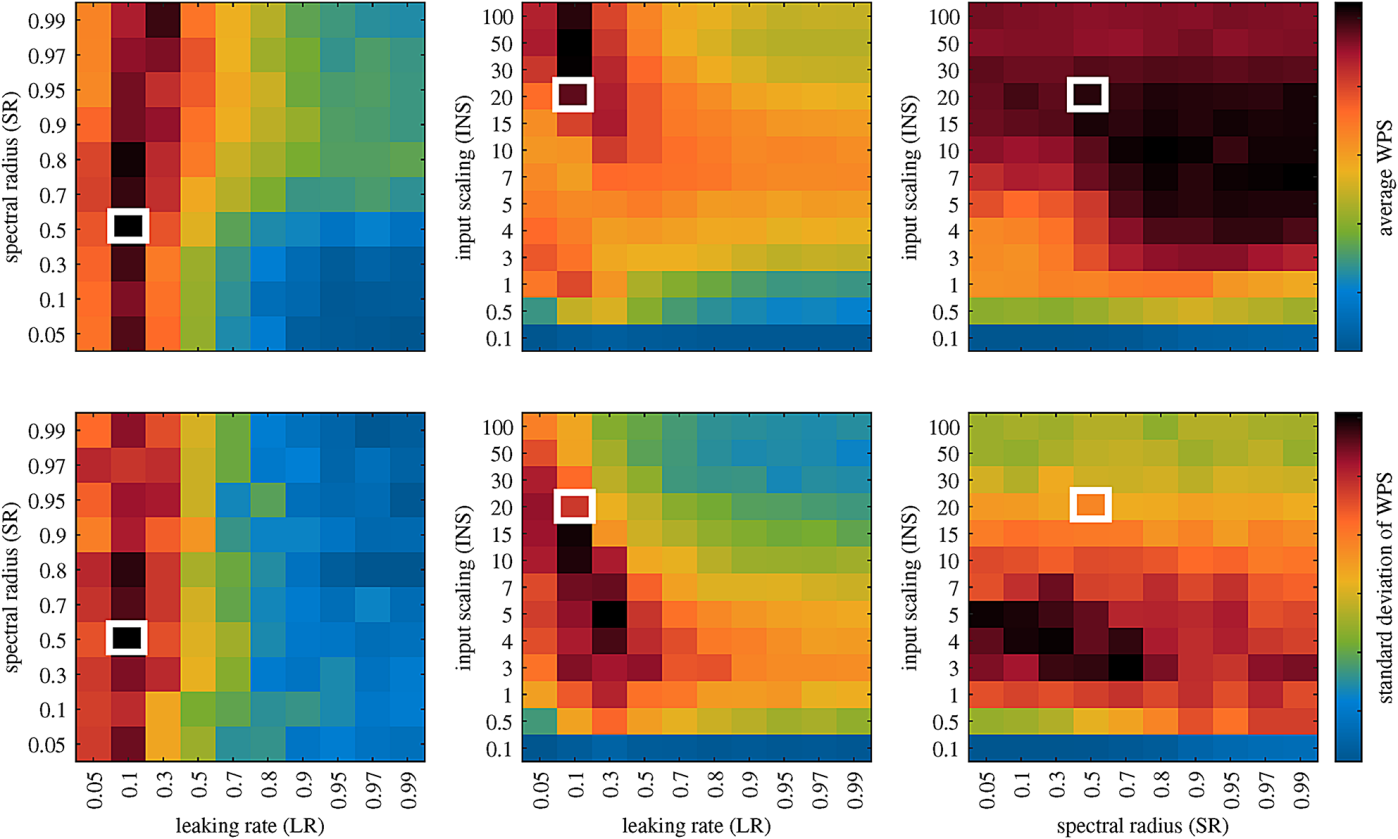
Matrix representation of the average WPS (top) along with the standard deviations (bottom) for the ESN predictions with respect to the hyperparameters. The highlighted squares show the best prediction set for LR = 0.1, SR = 0.5, and INS = 20. Please note that the axis scaling is not uniform for the various hyperparameter ranges. Figures were generated using MATLAB R2020b software (www.mathworks.com/products/new_products/release2020b.html).

The manuscript has so far focused on the similarity between the ground truth and the reconstructed reduced order model. Nevertheless, the variation of ESN performance with respect to its hyperparameters and random seed is also of concern and can provide valuable insights into their role in the reservoir’s performance and may help to apply them to other systems. The three main hyperparameters of the ESN, namely Leaking Rate (LR), Input Scaling (INS), and Spectral Radius (SR), along with the Random Seed (RS) of the reservoir have been taken as the variables that should be optimized for the ESN. Then, the ESN was optimized based on the Weighted Prediction Score (WPS), a variable to measure the degree of similarity between the prediction and ground truth (see Sec. 5 for further details) and also reasonable limits for the hyperparameters.

Figure 6 top shows the average values of the WPS for the entire 24 random seeds, and at the bottom are the standard deviations. The highlighted squares show the values of the best prediction. In general, a high mean WPS score with, at the same time, low standard deviation would be preferable. The high mean value is an indicator of a good model, whereas a low standard deviation indicates that the result is not too strongly dependent on the random initialization. Clearly, low LR values show better performance compared to higher LR values. Considering the fact that the LR is a representation of the neuron’s internal memory contribution, then low LR values mean a lower update speed of the system and more dependence on the previous state of the neuron. This is intuitively understandable, as most of the TCPMs of the flow have oscillations with very low frequencies (see Fig. 2b), and thus, fast updates of the neuron states inside the reservoir are not demanding. INS is the next variable, which has a relative trend, of course not as strong as LR, for the best predictions. It seems that as the contribution of input signals, which are basically the predicted output signals in the current closed-loop scenario, increases with the increase of INS value, the reservoir generally performs better. One can translate this to a very significant interdependency of the TCPMs to each other’s values. Finally, the reservoir performance seems to be affected by the SR values in a more complex way. Other than very low SR values ($<0.3$), for the rest of the range, it seems to be quite similar performance based on WPS. Although very high SR values (SR = 0.99) seem to have relatively better performance.

## Discussion

The current study conducted reduced order modeling of 3D turbulent Rayleigh-Bénard Convection (RBC) using an Echo State Network (ESN) with a reservoir of 1000 neurons. Proper Orthogonal Decomposition (POD) was performed on the experimental three-component velocity data in the horizontal mid-plane of an RB cell of aspect ratio 10 for 400 free fall times ($t_f \approx 3.5$ snapshots). The respective time coefficients of the first 100 POD modes (TCPM) were used as the signal waves for the ESN. The network was trained for 200 $t_f$ and reconstructed the flow for the next 200 $t_f$ in a closed-loop scenario. The results indicate that the ESN successfully reconstructs the dynamics of the flow in a way that makes it difficult to distinguish from the actual ground truth. It has been shown that this similarity goes beyond the oscillations of the TCPMs and is present in the statistics of the oscillations of the reconstructed velocity fields (spatial and temporal gradients), and even the corresponding vortex fields remain reasonable. This is quite substantial, assuming that the vortices represent energy dissipation in the flow. In other words, the ESN has been able to not only model the oscillations of the individual TCPMs, but also the interdependencies of the variation of the entire 100 modes used for the study. In terms of the hyperparameters, lower leaking rates (LR) and higher input scaling (INS) clearly showed better performance. This trend can be related to the underlying flow physics and may serve as an indicator for the design of ESN for other applications. However, the trend was less evident for the reservoir’s spectral radius (SR). The best performance in terms of the weighted prediction score was for INS = 20, SR = 0.5, and LR = 0.1.

The current study paves the way for the application of efficient machine learning methods in more turbulent flows. It displays the viability of reservoir computing in general and ESN in particular in dealing with complex examples of chaotic systems. Furthermore, it provides a scale for comparing the performance of other similar studies using more complex machine learning algorithms to see if there is really a benefit to the additional complexity. A possible direction for the expansion of the current study would be to apply a similar sort of modeling to the meteorological data due to the general similarity of the type of oscillations in RBC and atmosphere or use ESNs for subgrid modeling of these^35,36^. However, there is no doubt that subgrid scale modeling is a different path and whether ESN will be successful on that as well remains to be seen.

There is no doubt that the ESN can only model dynamics that it has been trained to model. But, the question is the boundary for the complexity of the system that the ESN can still model. Although comparing two chaotic systems in terms of complexity is not straight forward, however, one might consider the number of POD modes that are needed to capture certain ratio of the total kinetic energy as a measure. If so, recent studies on the dynamics of the global oceans show that around 30 first energetic modes capture 80 percent of the energy^37^, which is far less than the current 100 POD modes for RBC flow in the study. Therefore, one could argue that the global dynamics of the oceans and atmosphere through the year should have a same order of complexity as the task at hand and therefore it should be feasible to model them via ESNs.

## Methods

### Experimental data set

The nature of the turbulent flow of Rayleigh-Bénard convection is mainly identified by the Rayleigh (Ra) and Prandtl (Pr) numbers and the cell’s aspect ratio. $Ra = \alpha \Delta Tgh^3/\nu \kappa$, where $\alpha$ is the thermal expansion coefficient, $\Delta T = T_H-T_C$ is the temperature difference between heating and cooling plates, $\nu$ is the kinematic viscosity and $\kappa$ is the thermal diffusivity of the fluid. Higher Ra numbers mean a higher ratio of buoyancy-driven forces compared to friction and, thus, more turbulent flow. The Prandtl number is the ratio of momentum diffusivity to thermal diffusivity $Pr=\nu /\kappa$, and consequently, as Pr decreases the thermal diffusivity dominates the flow and thus the viscous boundary layer becomes thinner, and the thermal boundary layer becomes thicker, resulting in a more dynamic flow. For the current study, the RB cell has a height of 30 mm and a width of 300 mm, which results in an aspect ratio of 10. This is large enough to suppress the effect of side walls and create free movement of the superstructures in the flow. The cell is placed inside a pressure vessel^38^ (P = 2.5 bar) and filled with SF_6_ gas. The transparent heating plate of the cell has a temperature of $T_H=24.6^{\circ }C$ and the temperature difference of $\Delta T=6.9 K$ with the upper aluminum cooling plate. This results in $Ra = 5.5\times 10^6$ and $Pr = 0.79$. Although the flow data is experimental and independent of theoretical assumptions, however, in the current range of the pressure and density for SF_6_ gas, and $\Delta T=6.9$ K, the Boussinesq approximation is valid and also the radiation is negligible.

The flow has been seeded by DEHS (di(2-ethylhexyl) sebacate) particles of 1$\mu m$ diameter, and then the horizontal mid-plane of the cell has been illuminated by a double pulse laser. The transparent heating plate provides optical access to two cameras in order to conduct Stereoscopic Particle Image Velocimetry to reveal the three components of the velocity in the horizontal mid-plane. The data is recorded at 10 Hz frequency, which corresponds to 3.5 snapshots per free-fall time $t_f=h/\sqrt{\alpha \Delta Tgh}=0.35s$, and the field of view covers $A=7h\times 5.8h$ in the center of the cell. The final resolution of the data is $174\times 208$ vectors, which results in $1\times 1 {\textrm{mm}}^{2}$ of spatial resolution. Further details about the experimental setup and experimental data can be found in Ghazijahani et al.^38^ and Ghazijahani and Cierpka^29^, respectively.

### Echo state network

Fig. 1a right shows a schematic sketch of an Echo State Network (ESN). An ESN is a type of reservoir computing where only the output weights (red arrows) are trained^28,39^. The input signals are randomly connected to the neurons (n) inside the reservoir by a weight matrix of ${\hbox {W}}_{\textrm{in}}$ (blue arrows), and the neurons inside the reservoir are also randomly connected with a weight matrix named W (green arrows). Finally, each neuron is connected to its state in the previous time (s(n-1)) step by a coefficient equal to 1-LR (see equation 2 and 3). LR is the Leaking Rate of the reservoir and determines the update speed of the reservoir state; thus, it’s related to the dynamics of the system that the ESN encounters. Both W and ${\hbox {W}}_{\textrm{in}}$ are randomly preselected. The coefficients inside ${\hbox {W}}_{\textrm{in}}$are in the range of [± INS/2], and INS (Input Scaling) determines the contribution of the input signals, which are the output signals of the ESN in the previous time step for the closed loop scenario of the current study, on the reservoir dynamics. The maximum eigenvalue of the W is considered as Spectral Radius (SR). High values of SR represent more chaotic interactions between the reservoir neurons. However, the value of SR is advised to be lower than 1 in order to satisfy Echo State Property (ESP) for the reservoir. This ensures the independence of the reservoir from its initial state and unique dependence on the last inputs^27,39^.

2 $$\begin{aligned} \tilde{s}(n)= & {\tanh (W^{\textrm{in}}[1;u(n)]+Ws(n-1))} \end{aligned}$$

3 $$\begin{aligned} \ s(n)= & {(1-LR)s(n-1)+LR\tilde{s}(n)} \end{aligned}$$

Once the neuron states (s(n)) are calculated, the output weight matrix (${\hbox {W}}_{\textrm{out}}$) will be determined during the training phase as follows:

4 $$\begin{aligned} q(n)= & {W^{\textrm{out}}[1;u(n);s(n)]} \end{aligned}$$

5 $$\begin{aligned} W^{\textrm{out}}= & {\textrm{argmin}} \Biggl \{\frac{1}{N_\textrm{out}T} \sum \limits _{j=1}^{N_\textrm{out}} \Bigg [ \sum \limits _{i=1}^T ({q}_i(n)-q_i (n)^{\textrm{target}})^2+\beta \left\| {w}_j^{\textrm{out}}\right\| ^2 \Bigg ]\Biggl \} \end{aligned}$$

Here, $\beta>0$ is the ridge regression parameter, which prevents the amplification of small differences in state dimensions by large rows of ${\hbox {W}}_{\textrm{out}}$. In addition, it helps to prevent overfitting, in which the network performs excellently on the seen data but poorly on unseen data. For the current study, a reservoir with 1000 neurons is used. The network is trained for 700 time steps ($=200 t_f$) and predicts the next 700 time steps in a closed-loop scenario. The three main hyperparameters of the reservoir (LR, SR, and INS) along with the Random Seed (RS) that fixes the random state of the W and ${\hbox {W}}_{\textrm{in}}$are varied in order to find the best set of hyperparameters with the best performance. The optimization is performed based on Weighted Prediction Score (WPS) which is going to be described in the following section. The current ESN model has been written in Python using turbESN^40^library, which is inspired by the easyesn package^41^.

### Network optimization

Network optimization is highly dependent on the aim of the modeling and complexity of the dynamic system. Given the complexity of the oscillations of the TCPMs for the current 3D RBC flow, the aim is to reconstruct the system’s dynamics in a quality that makes it indistinguishable from the ground truth. Therefore, the aim is statistical convergence rather than exact deterministic alignment. However, this convergence is desired for the combination of the oscillations of all 100 TCPMs so that similar dynamics can emerge in the velocity field when they are all combined. Thus, assuming the predicted values from the ESN for mode i as $P = \{p_{i_1}, p_{i_2}, \ldots , p_{i_n}\}$ and the actual values as $A = \{a_{i_1}, a_{i_2}, \ldots , a_{i_n}\}$, Weighted Prediction Score (WPS) has been defined as the optimization parameter as follows:

6 $$\begin{aligned} \begin{aligned} WPS=&\sum \limits _{i=1}^{100} \Bigg [\times \Bigg (1-\frac{|(|{\bar{p}}_{i}|-|{\bar{a}}_{i}|)|}{max(|{\bar{p}}_{i}|,|{\bar{a}}_{i}|)}\Bigg )\times \Bigg (1-\frac{|({\sigma }_{p_i}-{\sigma }_{a_i})|}{max({\sigma }_{p_i},{\sigma }_{a_i})}\Bigg )\\&\times \Bigg (1-\frac{|({\sigma }_{p'_i}-{\sigma }_{a'_i})|}{max({\sigma }_{p'_i},{\sigma }_{a'_i})}\Bigg )\times E_i\Bigg ] \end{aligned} \end{aligned}$$

Here, the first part compares the absolute of the mean values of the oscillations ($\bar{p_i}$ and $\bar{a_i}$). Then, the second and third parts compare the standard deviation of the oscillations ($\sigma _{p_i}$ and $\sigma _{a_i}$) and derivatives of the oscillations ($\sigma _{p'_i}$ and $\sigma _{a'_i}$). Combining the mean, standard deviation, and standard deviation of derivatives is necessary since ignoring one of them can result in very similar WPS values for different oscillations. Finally, each mode is multiplied by its contribution to the total kinetic energy ($E_i$) in order to have a weighted contribution to the WPS based on the importance of the mode.

## Data Availability

The data that support the findings of this study have been deposited in the Springer Nature figshare repository: https://figshare.com/s/85584a86b6318363e220
